# Effects of Exercise Snack Program on Quality of Life, Cardiorespiratory Fitness, and Metabolic Flexibility in Elderly Cancer Survivors: A Preliminary Study

**DOI:** 10.3390/life15091401

**Published:** 2025-09-04

**Authors:** Peng Zhou, Zimei Hu, Taesung Kim, Yonghwan Kim, Zhengqing Leng, Moonyoung Choi

**Affiliations:** 1 School of Physical Education, Kashi University, Kashi 844000, China; 20251019@ksu.edu.cn (P.Z.); huzimei19891203@ksu.edu.cn (Z.H.); 2 Department of Physical Education, Gangneung-Wonju National University, Gangneung 25457, Republic of Korea; tsk1223@gwnu.ac.kr (T.K.); yhkim@gwnu.ac.kr (Y.K.); 3 Department of Physical Education, Daegu University, Gyeongsan 38453, Republic of Korea

**Keywords:** fitness, frail elderly, metabolic flexibility, training, post-cancer

## Abstract

Background: Cancer has a high mortality rate and leaves physical and mental difficulties even after treatment. When it afflicts frail elderly people, it poses a greater burden to them and society. Regular exercise helps to restore the deteriorated health of cancer survivors. The purpose of this study was to compare the effects of a short-term, high-repetition, home-based “exercise snack” program with those of a traditional, continuous, moderate-intensity exercise format on key health outcomes in elderly cancer survivors, including quality of life, cardiorespiratory fitness, and metabolic flexibility. Methods: A short-duration, high-repetition exercise snack group (ESG, n = 17) and a traditional exercise group (TEG, n = 17) were compared after 12 weeks of training. The effects of exercise on quality of life, cardiorespiratory fitness, metabolic flexibility, and blood lipids were measured. Results: Quality-of-life vitality, social functioning, cardiorespiratory fitness VO_2_ peak, and high-density lipoprotein cholesterol were improved more in ESG than in TEG (*p* < 0.05). Metabolic flexibility showed that fat utilization increased and carbohydrate utilization decreased post-training compared to pre-training at VO_2_ peak 20 and 40% in both groups (*p* < 0.05). Conclusions: In both groups, cardiorespiratory fitness was shown along with physical and psychological improvements in quality of life, and the efficiency of metabolic flexibility was also improved. Therefore, short-term, frequent exercise may be an appropriate exercise alternative for elderly people with difficulties.

## 1. Introduction

Cancer is one of the leading causes of death worldwide [1]. According to GLOBOCAN 2022 estimates, there were approximately 20.0 million new cancer cases and 9.7 million cancer-related deaths globally in 2022 [2]. Of these, 49.2% of all new cases and 56.1% of deaths occurred in Asia, despite the region comprising about 59.2% of the global population. Among various cancer types, stomach cancer remains particularly prevalent in East Asia [3]. In 2022, Korea ranked among the highest globally in age-standardized incidence rates for stomach cancer, with 32.7 cases per 100,000 people, compared to the global average of 11.1 [2]. This elevated incidence is attributed to disproportionately high case fatality rates and a greater prevalence of late-stage diagnoses in many Asian countries, underscoring significant regional disparities in cancer outcomes [3].

In recent decades, age-standardized cancer mortality rates have declined in many high-income and upper-middle-income countries, largely due to improved screening programs, earlier detection, and advances in treatment [4]. However, the psychosocial burden among survivors remains substantial, with many reporting persistent anxiety, fatigue, and concerns about recurrence and metastasis [5]. In addition to mental health challenges, long-term effects of cancer therapies—such as fatigue, appetite loss, and neuropathy—continue to impair quality of life (QoL). Such impairments can vary depending on cancer type and treatment modality, but are often pronounced in older survivors due to age-related vulnerability and reduced physiological resilience [6,7].

According to a study by Zamani et al., 19.1% of cancer survivors reported symptoms of anxiety, and 31.0% reported symptoms of depression, underscoring the importance of post-treatment psychosocial care [8]. Psychological distress is a common feature across cancer types, but may be amplified in survivors facing chronic treatment-related symptoms and reduced physical function [5]. Among elderly cancer survivors, sarcopenia and reductions in muscle strength are common and are associated with poor nutritional intake and prolonged recovery [9]. This functional decline contributes to reduced activity levels and a significant deterioration in overall health-related QoL [10].

Experts emphasize the importance of maintaining and restoring physical capacity and functionality after treatment, to improve QoL and mitigate long-term side effects. This necessitates multidisciplinary rehabilitation approaches that involve medical professionals, dietitians, and exercise specialists [11]. In particular, positive research on engaging in regular physical activity continues to be reported. In a study by Bausys et al., comprehensive application of endurance, respiratory muscle strength, stretching, and muscle strengthening exercises for 12 months improved walking ability and QoL in patients with cancer compared to the control group, while postoperative complications decreased [12]. According to a study by Kim et al., low-intensity and moderate-intensity exercises were performed daily for 30 min as home training, and a follow-up study was conducted after 1 year. The results showed that the short-term physical function score and the sit-to-stand score of the exercise group improved compared to those of the control group [13].

However, frail elderly people often face additional barriers to engaging in structured exercise programs, including comorbidities, environmental restrictions, and lack of accessibility [14]. These challenges are particularly severe among elderly individuals with limited mobility, as they are less likely to access rehabilitation centers or community fitness programs. Consequently, there is a persistent challenge in designing and validating accessible, effective exercise interventions for this population [15,16].

Recently, various exercise modalities have been proposed to overcome these accessibility issues. Among them, the “exercise snack” model—short bouts of physical activity performed multiple times throughout the day—has gained attention as a time-efficient and scalable approach. This model has been shown to improve cardiorespiratory fitness, muscular function, and metabolic flexibility in diverse populations, including older adults and clinical groups [17,18]. Fyfe et al. demonstrated the feasibility and acceptability of a home-based resistance exercise snack intervention in community-dwelling older adults, noting high adherence and positive effects on functional health [19]. These findings suggest that high-frequency, low-barrier interventions like exercise snacking are particularly relevant for older individuals who experience fatigue, mobility restrictions, or low motivation to engage in conventional programs. Given these characteristics, the exercise snack format may offer a more accessible and sustainable alternative to traditional exercise for frail, post-treatment older adults.

Therefore, this study aimed to compare the effects of an exercise snack program and traditional exercise participation in frail older adults after cancer treatment. QoL, blood lipid profiles, and cardiorespiratory fitness were selected as key indicators, as they reflect both subjective well-being and objective physiological recovery. In particular, cardiorespiratory fitness is a representative marker of physical fitness and a well-established predictor of healthy lifespan, especially in cancer survivors [20]. A study in adults diagnosed with cancer reported that individuals with high cardiorespiratory fitness had a 48% lower risk of cancer mortality and a 53% lower risk of all-cause mortality compared to those with lower fitness levels [21]. Impaired aerobic capacity and energy metabolism are commonly associated with fatigue, reduced functional independence, and poorer quality of life in this population [22]. Additionally, improvements in lipid metabolism and glycemic control are frequently mediated by enhanced aerobic capacity and metabolic flexibility [23]. Thus, measuring these domains together provides a multidimensional perspective on how exercise interventions impact physical, metabolic, and psychological health among elderly post-treatment cancer survivors.

The main objective of this study was to compare the effects of a short-term, high-repetition, home-based “exercise snack” program and a traditional, continuous, moderate-intensity exercise format in frail older adults after cancer treatment. The primary outcomes were QoL, cardiorespiratory fitness, and metabolic flexibility, which together reflect both subjective well-being and objective physiological recovery. The subsequent objective was to assess changes in substrate efficiency, defined as the capacity to effectively utilize lipids and carbohydrates during exercise and recovery [24]. In cancer survivors, treatment-related muscle loss, mitochondrial dysfunction, and reduced activity often impair this capacity, leading to greater carbohydrate reliance and lower lipid oxidation [25,26]. Such metabolic inflexibility contributes to fatigue, reduced endurance, and higher cardiometabolic risk [27]. Enhancing aerobic capacity through exercise can improve substrate efficiency by promoting mitochondrial adaptations and increasing lipid oxidation, thereby supporting recovery and long-term health [28]. Based on this rationale, we hypothesized that both exercise modalities would improve QoL, cardiorespiratory fitness, and substrate efficiency, with potential differences in the magnitude and pattern of these adaptations due to the distinct structural and physiological demands of each program.

## 2. Materials and Methods

### 2.1. Participants

This study was conducted with elderly men aged 65 years or older who were using a senior welfare facility and met the eligibility criteria relevant to the study objectives. Participants were eligible if they had previously been diagnosed with cancer and were capable of walking and engaging in daily activities independently but demonstrated signs of general frailty. Frailty was assessed based on clinical observation and inclusion criteria, rather than through the use of formal frailty indices. Participants were considered frail if they reported cancer-related fatigue, demonstrated reduced mobility, or had limitations in daily activities. These characteristics are consistent with key domains of widely accepted frailty models, including low physical activity, weakness, and unintentional weight loss [29].

To ensure that study outcomes could be attributed primarily to exercise interventions rather than to underlying medical conditions or pharmacological influences, rigorous exclusion criteria were applied during recruitment [30,31]. Participants were excluded if they had (1) severe mobility-limiting musculoskeletal conditions, such as Kellgren–Lawrence grade ≥3 knee osteoarthritis or a history of total knee arthroplasty; (2) unstable cardiovascular disease, including unstable angina, recent myocardial infarction within the past 6 months, decompensated heart failure, or uncontrolled hypertension (systolic ≥ 180 mmHg or diastolic ≥ 110 mmHg); (3) poorly controlled metabolic disorders, such as diabetes with HbA1c ≥ 9% or hyperglycemia with ketosis; (4) current pharmacological treatment for major depressive disorder; and (5) use of medications known to directly alter lipid or glucose metabolism, such as statins, insulin, or oral hypoglycemic agents, due to their potential to confound metabolic outcomes. Conversely, participants receiving stable, long-term treatments for conditions unlikely to influence the primary endpoints—such as well-controlled hypertension or benign prostatic hyperplasia—were considered eligible. This approach preserved ecological validity while minimizing bias from both health-related and pharmacological factors.

Sample size was estimated using G*Power software (version 3.1.9.4, University of Düsseldorf, Dusseldorf, Germany). With an effect size of f = 0.25, a power of 0.80, and an alpha level of 0.05, a total of 34 participants were allocated to either the exercise snack group (ESG) or the traditional exercise group (TEG) [32]. Group assignment considered participants’ mobility, exercise environment, health status, and individual preferences. A non-exercise control group was not included due to ethical considerations, as withholding physical activity in frail older cancer survivors was deemed inappropriate given the well-established risks associated with physical inactivity in this population. Therefore, the study focused on comparing two feasible and safe exercise modalities to evaluate their relative effectiveness in a real-world setting. All participants completed the 12-week program, underwent measurements relevant to the study objectives, voluntarily participated in all study procedures, and provided informed consent for participation. Cancer types among participants included stomach (n = 10), colorectal (n = 6), lung (n = 4), prostate (n = 3), liver (n = 2), thyroid (n = 7), lymphoma (n = 1), and pancreatic (n = 1). This study was approved by the Institutional Review Board of Gangneung-Wonju National University (Approval No. R202506).

### 2.2. Quality of Life Assessment

QoL was assessed using the 36-Item Short Form Health Survey (SF-36), a widely validated and reliable instrument for measuring health-related QoL in clinical and research settings [33]. The SF-36 comprises 36 items that evaluate eight domains of health: physical functioning, role limitations due to physical health, bodily pain, general health perceptions, vitality (energy/fatigue), social functioning, role limitations due to emotional problems, and mental health. These subdomains are conceptually grouped into two broader domains: physical health and mental health. Participants completed the questionnaire via self-administration using paper and pencil. Assistance was provided only when participants explicitly requested clarification or support in understanding specific items. All assessments were supervised by trained research staff with academic backgrounds in geriatric health and exercise science. Prior to data collection, the assessors received standardized instruction in questionnaire administration and response verification protocols to ensure consistency and minimize response bias. The Likert-type response format captured the extent of perceived limitations, symptom severity, and emotional well-being, with higher scores indicating better health status. The SF-36 was selected for its ability to comprehensively reflect the multidimensional impact of cancer and its treatment on both physical and mental health, particularly in older adult populations.

### 2.3. Cardiorespiratory Fitness and Metabolic Flexibility

Cardiorespiratory fitness and metabolic flexibility were assessed using a graded exercise test (GXT), conducted according to the American College of Sports Medicine (ACSM) guidelines with a modified Bruce protocol [34]. The test was performed on a Vmax29 cardiopulmonary exercise testing system (SensorMedics Co., Yorba Linda, CA, USA) using the standard Bruce protocol. All tests were conducted under the supervision of a licensed physician and a certified clinical exercise physiologist. Continuous 12-lead electrocardiogram (ECG) monitoring was employed to observe cardiac responses, and heart rate, blood pressure, and ECG signals were continuously recorded. The ratings of perceived exertion (RPE) and chest discomfort scores were documented every 3 min. The test was terminated upon reaching an RPE ≥ 17, through voluntary cessation by the participant, or due to clinical indicators requiring discontinuation (e.g., abnormal ECG, hypertensive response, or reported chest pain). Data from tests terminated for medical reasons were excluded from the final analysis. Oxygen uptake was measured on a breath-by-breath basis, and peak oxygen consumption (VO_2_ peak) was expressed relative to body mass (mL/kg/min). Anaerobic threshold (AT) was identified at the point of a sustained nonlinear increase in ventilatory volume, based on 10 s averaged values, and was expressed as a percentage of VO_2_ peak. To evaluate metabolic flexibility, substrate utilization patterns were estimated by analyzing the respiratory gas exchange ratio (RER) and calculating rates of fat and carbohydrate oxidation using indirect calorimetry. VO_2_ and VCO_2_ values were collected at each exercise stage and used in substrate-specific oxidation equations previously validated by Yang et al. [35].

### 2.4. Blood Lipid–Cardiovascular Disease Risk Factors

To evaluate changes in cardiovascular disease risk, a panel of metabolic biomarkers—total cholesterol (TC), low-density lipoprotein cholesterol (LDL-C), high-density lipoprotein cholesterol (HDL-C), triglycerides (TG), and fasting blood glucose—was assessed at baseline and after the 12-week intervention [36]. Participants were instructed to fast for at least 8 h prior to each measurement. Venous blood samples were collected from the antecubital vein in the morning between 08:00 and 09:30 to minimize circadian variation. All samples were analyzed by a certified clinical laboratory using standardized enzymatic colorimetric assays on an automated analyzer (Hitachi 747, Hitachi High-Technologies, Tokyo, Japan). Quality control procedures were conducted daily according to laboratory protocols.

### 2.5. Exercise Interventions

#### 2.5.1. Exercise Snack Program

**Figure 1:**
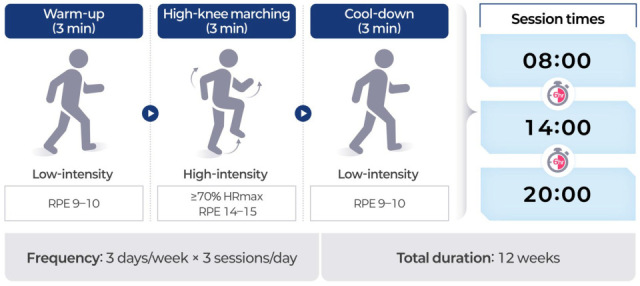
Structure and schedule of the exercise snack protocol.

Participants assigned to the exercise snack group (ESG) performed a time-efficient, distributed physical activity regimen designed to elicit cardiovascular and metabolic benefits while accommodating the functional limitations common among older adults and cancer survivors. The selected protocol aligns with the operational definition of an “exercise snack,” which refers to short bouts of moderate-to-vigorous-intensity activity performed multiple times per day, allowing for accumulated physiological benefits without requiring long continuous sessions. This approach has been proposed as an effective and feasible strategy for improving health outcomes in populations with low exercise tolerance or limited time availability [17]. The intervention was conducted over 12 weeks, with participants completing three sessions per day, approximately 6 h apart (e.g., 08:00, 14:00, and 20:00), three days per week. Each session lasted a total of 9 min, consisting of a 3 min warm-up, a 3 min high-intensity bout, and a 3 min cool-down (Figure 1). The warm-up and cool-down phases involved low-intensity movements such as walking in place, performed at a self-perceived exertion of 9–10 on the Borg 6–20 scale. The high-intensity component, which served as the central stimulus of each session, consisted of high-knee marching in place. Participants were instructed to swing both arms vigorously in coordination with each knee lift, maintaining a full range of motion through the shoulders and elbows. The simultaneous engagement of the upper and lower limbs was intended to elevate heart rate response and metabolic demand, thereby maximizing the physiological stimulus of the high-knee exercise [37]. Participants were instructed to raise their knees to at least hip height and to maintain a cadence of 100 to 110 steps per minute [38]. A metronome application was used to ensure consistency of pace. Exercise intensity was individualized to target ≥ 70% of maximal heart rate (HRmax), calculated using the standard age-predicted formula (HRmax = 220 − age). RPE was also monitored using the Borg scale, with values between 14 and 15 reflecting vigorous-intensity effort. This intensity range was selected based on studies demonstrating the safety and efficacy of similar workloads in older adults [39]. In addition, participants were encouraged to adjust their effort within the prescribed repetition and intensity ranges to accommodate daily variations in physical capacity, ensuring that each exercise bout remained both challenging and achievable. The exercise snack protocol was implemented as a home-based intervention to promote accessibility and minimize logistical barriers. All participants received individualized in-person orientation sessions prior to program initiation, during which they were trained on proper exercise technique, use of the metronome app, and heart rate monitor operation. These sessions were delivered by certified exercise specialists trained in geriatric fitness. Participants performed the exercises at home and were provided with printed instructions and daily log sheets. To ensure compliance and safety, weekly check-ins were conducted remotely via phone or video calls by trained research staff. These calls included review of heart rate data, RPE values, and self-reported exercise logs. This hybrid structure allowed for effective delivery of a home-based regimen while maintaining fidelity to the intended intensity and structure of the protocol.

#### 2.5.2. Traditional Exercise Program

Participants assigned to the traditional exercise group (TEG) engaged in a structured aerobic training program designed to reflect the American College of Sports Medicine’s recommendations for cancer survivors and older adults [39]. The program emphasized continuous, moderate-intensity physical activity, with the primary objective of improving cardiorespiratory fitness and metabolic health through sustained aerobic effort. All exercise sessions were led by nationally certified exercise professionals with clinical experience in geriatric populations. Each instructor had completed formal training in exercise prescription for older adults and supervised the program in accordance with safety protocols established by the welfare facility. The intervention was conducted over 12 weeks, with participants completing three exercise sessions per week on nonconsecutive days (e.g., Monday, Wednesday, and Friday). Each session lasted 30 min and was performed as a continuous walking exercise on a motorized treadmill under indoor conditions. This mode of aerobic training was selected for its practicality, safety, and effectiveness in delivering consistent cardiovascular stimulus with minimal orthopedic strain. Participants were instructed to maintain a target exercise intensity corresponding to 40–60% of HRmax, calculated using the standard age-predicted formula. Exercise intensity was monitored in real time using wrist-worn heart rate monitors. In addition, RPE values were recorded using the Borg 6–20 scale, with participants encouraged to remain within a range of 11 to 13, corresponding to moderate intensity. To ensure proper pacing and safety, each session was supervised by trained research staff, who provided guidance on treadmill speed adjustments and monitored for signs of fatigue, dizziness, or abnormal cardiovascular responses. Each session included a 5 min warm-up at a self-selected low walking speed and a 5 min cool-down period during which speed was gradually reduced. The 20 min main phase of moderate-intensity treadmill walking was conducted at a steady pace, individualized to each participant based on baseline fitness and tolerance. Participants maintained daily exercise logs that documented session completion, average heart rate, treadmill speed, and perceived exertion. This protocol was designed to serve as a standard comparison condition against the exercise snack intervention, ensuring that both groups received equivalent weekly exercise exposure in terms of total duration and cumulative cardiovascular load.

#### 2.5.3. Comparison of Exercise Protocols

Although both exercise protocols were developed in accordance with the ACSM guidelines for cancer survivors and older adults [34], they differed in terms of prescribed intensity and structural delivery. The ESG protocol emphasized high-frequency, short-duration bouts of vigorous-intensity exercise (≥70% HRmax, RPE 14–15), while the TEG protocol utilized longer, continuous sessions at moderate intensity (40–60% HRmax, RPE 11–13). Both groups exercised for a comparable total weekly duration (81 min for ESG vs. 90 min for TEG), and intensity was monitored using heart rate tracking and RPE logs. However, direct energy expenditure (e.g., total kcal or MET-min) was not assessed. Therefore, although the protocols were matched in terms of total time and targeted exertion zones, they cannot be considered physiologically equivalent. Rather, the study aimed to compare two distinct aerobic training strategies—differing in structure and relative intensity—but each aligned with recommended thresholds for promoting health in this population.

### 2.6. Data Analysis

Statistical analysis was performed using SPSS version 25.0 (IBM Corp., Armonk, NY, USA). A Shapiro-Wilk test was used to test for normality, and this study followed the parametric method. To compare TEG and ESG, continuous variables were tested using an independent *t*-test, and categorical variables were tested using a chi-square test. Repeated two-way ANOVA was used to examine the interaction effect between groups and time, and post hoc analyses were conducted to determine the significance of the pre-post test. In the comparison between groups, Cohen’s d and Cramer’s V were used for effect size. The significance test level was *p* < 0.05.

## 3. Results

### 3.1. General Characteristics

General characteristics were compared between groups (Table 1). There were no significant differences between groups in age, height, weight, BMI, blood pressure, or heart rate. In addition, there were no differences between groups in cancer recurrence or metastasis, education level, or occupation. However, there was a significant difference in TEG 4.5 ± 2.5 vs. ESG 6.8 ± 3.9 in the year after cancer treatment. Both groups demonstrated high adherence to their assigned exercise interventions. Adherence rates, calculated as the proportion of completed sessions relative to the prescribed total, were 94.1% in ESG and 92.6% in TEG.

### 3.2. Quality of Life Change

**Figure 2:**
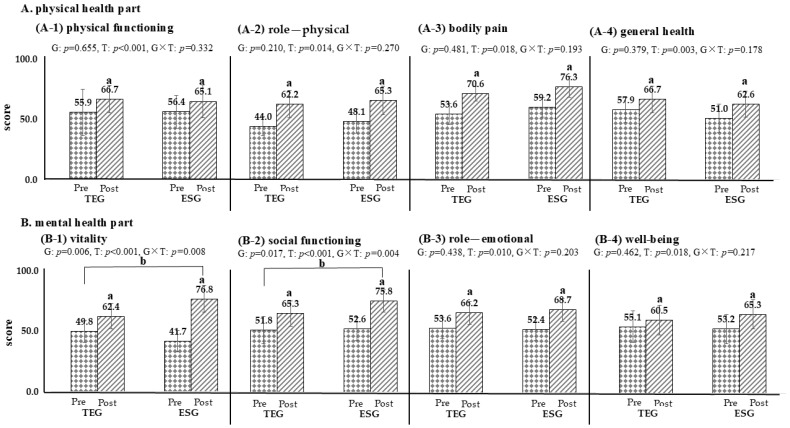
Comparison of changes in quality of life based on exercise groups (TEG, n = 17; ESG, n = 17). *p* < 0.05; a, pre-post comparison within the group; b, interactions based on time and groups; TEG, traditional exercise group; ESG, exercise snack group; G, group; T, time. The dot is Pre, the diagonal line is Post. (**A-1**) physical functioning; (**A-2**) role—physical; (**A-3**) bodily pain; (**A-4**) general health; (**B-1**) vitality; (**B-2**) social functioning; (**B-3**) role—emotional; (**B-4**) well-being.

Following the 12-week intervention, both ESG and TEG demonstrated significant improvements across multiple domains of the SF-36 (Figure 2). In the physical health domains—including physical functioning, role limitations due to physical health, general health perceptions, and bodily pain—both groups showed statistically significant within-group improvements (*p* < 0.05), with no significant differences observed between groups. In the mental health domains—comprising vitality, social functioning, role limitations due to emotional problems, and mental health—significant improvements were also observed in both groups (*p* < 0.05). Notably, vitality and social functioning scores improved to a significantly greater extent in ESG than in TEG (*p* < 0.05), indicating domain-specific benefits associated with the exercise snack intervention.

### 3.3. Cardiorespiratory Fitness

**Figure 3:**
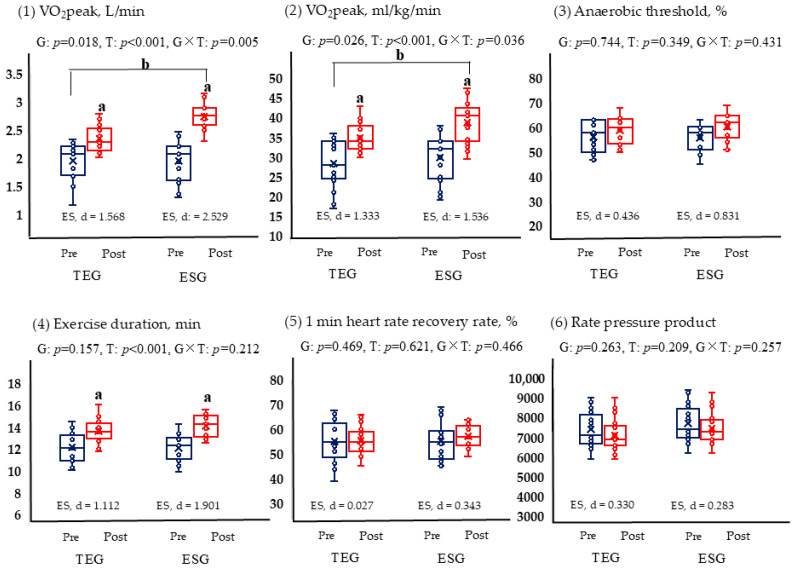
Comparison of changes in graded exercise test results based on exercise (TEG, n = 17; ESG, n = 17). *p* < 0.05; a, pre-post comparison within the group; b, interactions based on time and groups; TEG, traditional exercise group; ESG, exercise snack group; G, group; T, time. The blue is pre, the red is post. (**1**) VO2peak, L/min; (**2**) VO2peak, ml/kg/min; (**3**) Anaerobic threshold, %; (**4**) Exercise duration, min; (**5**) 1 min heart rate recovery rate, %; (**6**) Rate pressure product.

Following the 12-week intervention, both ESG and TEG exhibited significant improvements in VO_2_ peak (*p* < 0.05) (Figure 3). Between-group analysis indicated that ESG achieved a significantly greater increase in VO_2_ peak compared to TEG (*p* < 0.05). Exercise duration also increased significantly in both groups (*p* < 0.05), although no significant difference was observed between the groups. In contrast, AT, 1 min heart rate recovery, and rate–pressure product values did not change significantly in either group, and no between-group differences were detected.

### 3.4. Metabolic Flexibility

**Figure 4:**
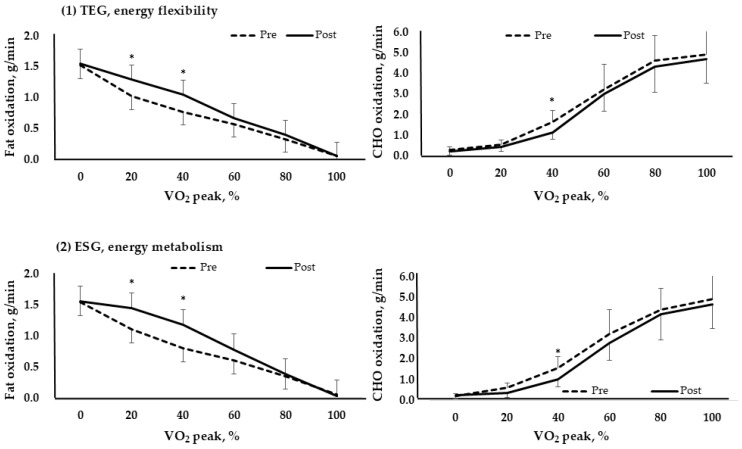
Fat and carbohydrate metabolism based on VO_2_ peak % in each group (TEG, n = 17; ESG, n = 17). *, *p* < 0.05; TEG, traditional exercise group; ESG, exercise snack group; CHO, carbohydrate. (**1**) TEG, energy flexibility; (**2**) ESG, energy metabolism.

Figure 4 compared the fat and carbohydrate usage by exercise intensity (VO_2_ peak%) for each group pre and post. At low intensity, fat is mainly used, and as the intensity gradually increases, carbohydrate usage increases. The results showed that fat usage increased at 20% and 40% for both TEG and ESG (*p* < 0.05). Furthermore, carbohydrates significantly decreased at 40%. This means that efficiency increased by promoting fat oxidation (*p* < 0.05).

### 3.5. Cardiovascular Disease Risk Factors

Following the 12-week intervention, both ESG and TEG demonstrated significant within-group reductions in TG and fasting glucose levels (*p* < 0.05) (Table 2). HDL-C increased significantly in ESG (*p* < 0.05), whereas no significant change was observed in the TEG. No statistically significant changes were found in LDL-C or TC in either group. Between-group comparisons revealed a significantly greater reduction in TG levels in TEG compared to ESG (*p* < 0.05), while the increase in HDL-C was significantly greater in ESG than in TEG (*p* < 0.05). No significant between-group differences were observed for fasting glucose, LDL-C, or TC.

## 4. Discussion

This study compared the effects of two distinct aerobic training modalities, a distributed “exercise snack” protocol and a traditional continuous format, both applied in frail older adults following cancer treatment. These interventions were designed to differ in structure and delivery setting, allowing an examination of how variations in frequency, intensity, and session duration influence outcomes related to quality of life, aerobic capacity, and metabolic function. While both programs led to positive changes, the observed improvements varied across outcome domains. These differences likely reflect the specific physiological and behavioral demands of each intervention. The exercise snack protocol, characterized by brief, high-frequency sessions performed at home, appeared to be more effective in enhancing perceived vitality and mental well-being. In contrast, the traditional format, which involved longer supervised sessions, resulted in greater gains in cardiorespiratory fitness. These findings are consistent with previous research suggesting that the structure and accessibility of exercise programs are important determinants of health outcomes in older populations with functional limitations [19].

Building on these general findings, the observed changes in cardiovascular disease risk markers warrant closer examination. Both groups exhibited significant reductions in fasting triglyceride and glucose levels following the 12-week intervention; however, the pattern and magnitude of these changes differed depending on the exercise modality. Participants in TEG demonstrated a significantly greater reduction in triglyceride levels compared to those in ESG. This finding aligns with prior evidence suggesting that continuous, moderate-intensity aerobic exercise enhances skeletal muscle lipoprotein lipase (LPL) activity, thereby facilitating triglyceride clearance via increased fatty acid uptake and oxidation [40]. While LPL activity may indirectly influence hepatic very-low-density lipoprotein (VLDL) secretion, this effect is likely mediated through broader metabolic adaptations, including improved insulin sensitivity and reduced hepatic lipid availability [41]. The sustained format of the TEG protocol—characterized by longer bout duration and uninterrupted energy demand—may have provided a more robust lipid-lowering stimulus. Conversely, a significantly greater increase in HDL-C was observed in ESG. Although such improvements are traditionally associated with prolonged aerobic training, emerging research indicates that intermittent physical activity—particularly when performed at moderate-to-vigorous intensities—can beneficially influence HDL-C concentrations via mechanisms involving reverse cholesterol transport and endothelial shear stress-mediated vascular adaptations [42]. The high-frequency structure of the ESG protocol, which incorporated full-body movement patterns multiple times daily, may have delivered repeated metabolic stimuli sufficient to induce these effects, despite the brief duration of each session.

Both groups also demonstrated significant reductions in fasting glucose levels, though no between-group differences were detected. This suggests that total aerobic workload, rather than the specific distribution of sessions, may be the predominant driver of glycemic improvement. Aerobic exercise enhances glucose uptake in skeletal muscle through insulin-independent mechanisms, particularly via AMP-activated protein kinase (AMPK) activation and GLUT4 translocation [43]. Although ESG and TEG differed in intensity and session format, the total weekly time engaged in purposeful aerobic activity was of a comparable order of magnitude, which may have provided a sufficient cumulative stimulus in both groups to elicit similar glucose-lowering responses. No significant changes were observed in LDL-C or TC levels in either group. These findings are in line with prior meta-analyses indicating that moderate-intensity aerobic exercise, when undertaken without concurrent dietary intervention or substantial changes in body composition, exerts limited influence on LDL-C concentrations [44]. As both interventions in the present study focused exclusively on aerobic exercise without modifications to caloric intake or macronutrient composition, the absence of significant change in these lipid parameters likely reflects the metabolic threshold required to elicit LDL-C reductions.

Changes in health-related QoL following the intervention revealed significant and broad-based improvements across both physical and mental health domains. In line with prior findings that link regular aerobic activity to enhanced functional status and symptom management in cancer survivors [45,46], both groups exhibited meaningful gains in physical functioning, general health perceptions, role limitations due to physical problems, and bodily pain. In the mental health-related domains, both ESG and TEG participants reported significant improvements in vitality, social functioning, emotional role limitations, and mental health status. These findings align with existing evidence that aerobic exercise—through modulation of neuroendocrine function and psychosocial engagement—can exert beneficial effects on mood, energy, and social participation [47,48]. Notably, ESG demonstrated significantly greater improvements in vitality and social functioning compared to TEG. These domain-specific differences may be attributed to the distributed, high-frequency structure of the exercise snack protocol, which may enhance perceived daily energy, foster a sense of accomplishment through repeated completion, and promote behavioral activation across multiple time points during the day [49,50].

The behavioral science literature suggests that repeated exposure to self-paced, achievable tasks can reinforce intrinsic motivation and emotional engagement, aligning with the core tenets of Self-Determination Theory, particularly autonomy and competence support [49]. Additionally, from a social cognitive perspective, high-frequency, low-barrier exercise routines may lower perceived effort thresholds and reduce avoidance tendencies, thereby enhancing self-efficacy and supporting long-term behavioral adherence. Beauchamp et al. reported that consistent exposure to achievable physical activity tasks within a self-regulated environment can reinforce confidence in one’s ability to exercise, decrease psychological resistance, and strengthen the internalization of behavioral goals, all of which are central to sustained engagement within the Social Cognitive Theory framework [50]. Taken together, these results suggest that while both aerobic modalities are effective in enhancing QoL among older cancer survivors, the exercise snack approach may confer additional advantages in domains sensitive to energy availability and social functioning.

Changes in cardiovascular fitness following the 12-week intervention revealed notable improvements in aerobic capacity across both exercise groups. Significant increases in VO_2_ peak were observed in ESG and TEG, aligning with a robust body of literature demonstrating that structured aerobic training can enhance maximal oxygen uptake (VO_2_ max) in older adults, including those with a history of cancer treatment [51,52]. Notably, ESG exhibited a significantly greater increase in VO_2_ peak than TEG. This may reflect the metabolic and neuromuscular benefits of repeated, high-frequency engagement of large muscle groups, as facilitated by the distributed structure of the exercise snack protocol. Prior studies suggest that frequent, moderate-to-vigorous activity bouts can yield meaningful cardiovascular adaptations by increasing peripheral oxygen extraction, stroke volume, and mitochondrial function, even in the absence of prolonged continuous exertion [53,54]. Jenkins et al. observed that a six-week stair-climbing exercise snack intervention resulted in an approximate 5% increase in VO_2_ peak and a 12% improvement in peak power output among sedentary young adults, despite the minimal cumulative exercise volume [53]. Similarly, a randomized controlled trial by Yin et al. demonstrated that six weeks of stair-climbing exercise snacks led to a ~7% increase in VO_2_ peak in inactive adults, whereas a time-matched moderate-intensity continuous training protocol failed to induce significant improvements [54]. Taken together, these findings underscore the efficacy of brief, distributed high-intensity stimuli in enhancing cardiorespiratory fitness and neuromuscular function through repeated peripheral muscle activation and transient elevations in metabolic demand.

Exercise duration during testing also increased significantly in both groups, indicating enhanced endurance performance. However, no between-group differences were observed, suggesting that improvements in time to exhaustion may be attributable to the overall cumulative aerobic stimulus rather than the specific structure or intensity distribution of the sessions. Although ESG and TEG differed in training format, the total weekly time spent performing purposeful aerobic exercise was of a similar order of magnitude, potentially providing a sufficient cumulative stimulus to produce comparable endurance gains in both groups. In contrast, no significant changes were observed in AT values in either group. This finding is consistent with previous evidence indicating that moderate-intensity aerobic training, particularly when performed at or below the AT, induces minimal adaptations in submaximal metabolic thresholds [55]. A meta-analysis by Goncalves et al. examined the effects of moderate-intensity training in patients undergoing cardiac rehabilitation [55]. The findings revealed significant improvements in VO_2_ peak but no corresponding changes in AT, suggesting that exercise intensity below the ventilatory threshold may be insufficient to induce meaningful adaptations in AT-related physiological parameters. ESG’s intermittent but non-maximal exertion levels and TEG’s steady-state protocol likely fell below the stimulus threshold required for substantial AT adaptation. These results underscore the importance of exercise intensity prescription when targeting submaximal metabolic thresholds and suggest that higher-intensity or interval-based interventions may be necessary to elicit meaningful changes in AT among deconditioned individuals.

The 1 min heart rate recovery measures the heart’s recovery rate, and a rapid return to resting after exercise is interpreted positively [56]. Furthermore, the rate–pressure product, calculated by multiplying heart rate and systolic blood pressure, is a way to measure cardiac workload [57]. The effect of training is interpreted as a lower value indicating cardiac health in the same resting condition. Although neither of these variables showed significant results in this analysis, this may be due to the training period not being long enough to expect significant changes.

RER represents the ratio of carbohydrate to fat burned according to exercise intensity. Typically, RER ranges from 0.7 to 1.0. The low-intensity range, where fat is burned most, is 0.7, while the high-intensity range, where carbohydrates are burned most, is 1.0. A positive change in RER as a result of training is a decrease in RER during exercise of the same intensity, indicating an increase in the ratio of fat burned to carbohydrates [26].

From this perspective, changes in substrate oxidation patterns observed during graded exercise testing provide evidence of favorable adaptations in skeletal muscle metabolic flexibility following the 12-week intervention. Both ESG and TEG exhibited increased fat oxidation and reduced carbohydrate reliance during low-to-moderate-intensity exercise (20% and 40% VO_2_ peak), reflecting enhanced metabolic flexibility—a key physiological trait characterized by the ability to appropriately shift between lipid and glucose utilization based on energy demands [35]. While this general pattern has been previously documented in endurance-based training, the current study extends these findings to older digestive cancer survivors [58], a population in which metabolic impairments are frequently reported.

Between-group comparisons revealed that TEG elicited significantly greater absolute fat oxidation at 40–60% VO_2_ peak compared to ESG. This difference aligns with the physiological adaptations induced by continuous moderate-intensity aerobic training, which is known to enhance fatty acid oxidation capacity within the range of 45–65% VO_2_ max—the intensity window where maximal fat oxidation (MFO) typically occurs [59]. These adaptations include increased capillary density, elevated expression of fatty acid transport proteins such as CD36, and enhanced intramuscular triacylglyceride (IMTG) mobilization through hormone-sensitive lipase (HSL) activity, collectively contributing to greater reliance on lipid metabolism during exercise in the “fat-burning zone” [60]. Importantly, no significant between-group differences in carbohydrate oxidation were observed during exercise, suggesting that while lipid metabolism adaptations differed by training modality, the capacity for glucose utilization remained comparable. This may reflect the matched aerobic training volume between groups, supporting the hypothesis that total energy expenditure plays a contributing role in modulating exercise-induced carbohydrate oxidation capacity [61]. Taken together, these findings indicate that both continuous and distributed aerobic exercise formats can improve metabolic flexibility in older cancer survivors, with modality-specific adaptations reflecting the physiological demands of each protocol.

Although this study was conducted on elderly cancer survivors, it has the following limitations. Although nutrition and habitual physical activity are important factors influencing the health outcomes of older adults and cancer survivors, these variables were not systematically measured in the present study. This omission was primarily due to resource limitations and the need to minimize participant burden in a frail population. While general dietary guidance was provided as part of routine care, individualized nutritional intake and daily physical activity outside of the intervention could not be quantified, which may have introduced residual confounding. Future research should incorporate comprehensive dietary assessment and objective physical activity monitoring to improve the accuracy of interpreting exercise intervention effects. The disease history and health status of the elderly are very diverse, and since measurements took place at a single institution, the results of this study may not be consistent with those in other environments and conditions; moreover, the researchers’ bias may be involved due to the convenience sampling method, and the representativeness of the data is limited. In addition, there was a significant difference between groups in the mean years since completion of cancer treatment. While this factor could potentially influence recovery trajectories in cancer survivors, all participants met the same eligibility criteria for functional status and readiness for exercise at baseline, which may have limited its impact on the observed outcomes. Nonetheless, future studies should consider stratifying participants or statistically adjusting for this variable to ensure more precise interpretation of intervention effects. Furthermore, although the SF-36 includes domains related to social functioning, this study did not incorporate direct measures of social context or interpersonal connectedness. Given the home-based nature of the intervention, the lack of formal assessment in this area limits our ability to fully interpret improvements in psychosocial outcomes. Future studies should consider integrating validated tools to capture social interaction and support networks more precisely. As only elderly men were included, future research on elderly women, a group that is likely to be excluded from health services, will be necessary.

## 5. Conclusions

This study compared the effects of an exercise snack protocol and a traditional continuous aerobic training model on cardiorespiratory fitness, metabolic flexibility, and health-related QoL in older adults following digestive cancer treatment. Both interventions produced meaningful improvements in aerobic capacity, substrate oxidation efficiency, and QoL outcomes, indicating that structured aerobic exercise can serve as a beneficial component of survivorship care in this population. Importantly, the exercise snack protocol elicited greater improvements in VO_2_ peak and selected QoL domains—particularly vitality and social functioning—despite its brief and distributed format. Meanwhile, the traditional exercise protocol led to superior fat oxidation during submaximal exertion, consistent with adaptations to sustained aerobic workload. These findings suggest that both exercise modalities offer distinct physiological advantages. Integrating flexible, home-based aerobic strategies may enhance accessibility and support physical recovery in older cancer survivors with functional limitations.

## Figures and Tables

**Table 1:** Sociodemographic characteristics of participants (TEG, n = 17; ESG, n = 17).

Variables	TEG (n = 17)	ESG (n = 17)	*t* or χ^2^	Effect Size	*p*-Value
Age, years	69.3 ± 3.2	68.5 ± 3.1	0.762	0.253	0.452
Height, cm	165.6 ± 6.7	165.0 ± 6.1	0.275	0.009	0.785
Weight, kg	69.2 ± 8.1	67.6 ± 6.9	0.611	0.212	0.545
Body mass index, kg/m^2^	25.2 ± 2.2	24.8 ± 2.8	0.269	0.595	0.267
Resting systolic BP, mmHg	121.3 ± 4.1	122.1 ± 4.3	1.324	0.285	0.687
Resting diastolic BP, mmHg	80.6 ± 5.0	81.3 ± 3.2	−0.278	0.166	0.781
Resting heart rate, beat	64.7 ± 3.9	66.9 ± 3.7	−0.496	0.578	0.620
Post-cancer, year	4.5 ± 2.5	6.8 ± 3.9	−2.120	0.726	0.042
Exercise adherence, %	94.1 ± 3.2	92.6 ± 3.0	0.348	0.483	0.385
Recurrence or metastasis, *n* (%)					
No	13 (76.5%)	15 (88.2%)	0.810	0.154	0.656
Yes	4 (23.5%)	2 (11.8%)			
Education, *n* (%)					
to middle	11 (64.7%)	12 (70.6%)	0.186	0.074	0.911
to high	4 (23.5%)	3 (17.6%)			
above college	2 (11.8%)	2 (11.8%)			
Occupation, *n* (%)					
No	11 (64.7%)	13 (76.5%)	0.567	0.452	0.708
Yes	6 (35.3%)	4 (23.5%)			

*p* < 0.05; TEG, traditional exercise group; ESG, exercise snack group; BMI, body mass index; BP, blood pressure.

**Table 2:** Lipid risk factor of cardiovascular disease (TEG, n = 17; ESG, n = 17).

Variables	Group	Pre	Post	*Df*, %	Pre-Post *p*-Value	*p*-Value
TC, mg/dL	TEG	169.2 ± 10.3	153.9 ± 18.8	−9.0	0.332	G: 0.303, T: 0.067 G × T: 0.507
	ESG	157.9 ± 18.8	152.4 ± 17.7	−3.5	0.425	
HDLC, mg/dL	TEG	42.6 ± 9.7	44.6 ± 8.4	4.7	0.213	G: 0.011, T: 0.006 G × T: 0.018
	ESG	43.1 ± 4.7	49.9 ± 5.9	15.8	<0.001	
LDLC, mg/dL	TEG	133.6 ± 29.1	125.6 ± 27.3	−6.1	0.180	G: 0.653, T: 0.201 G × T: 0.685
	ESG	130.9 ± 21.1	123.1 ± 64.8	−6.0	0.241	
TG, mg/dL	TEG	168.6 ± 19.6	136.6 ± 16.7	−19.0	0.003	G: 0.05, T: <0.001 G × T: 0.012
	ESG	164.4 ± 13.5	147.3 ± 10.6	−10.4	0.018	
Glucose, mg/dL	TEG	121.9 ± 18.8	110.3 ± 16.6	−9.5	<0.001	G: 0.621, T: 0.013 G × T: 0.165
	ESG	125.6± 16.2	109.1 ± 11.1	−13.1	0.004	

*p* < 0.05; TEG, traditional exercise group; ESG, exercise snack group; G, group; T, time; TC, total cholesterol; HDLC, high-density lipoprotein cholesterol; LDLC, low-density lipoprotein cholesterol; TG, triglycerides.

## Data Availability

The data presented in this study are available on reasonable request from the corresponding author.
